# Loneliness and burnout among doctors in a public academic hospital in South Africa

**DOI:** 10.4102/sajpsychiatry.v32i0.2675

**Published:** 2026-10-23

**Authors:** Sarveshwaree Kissoon, Jacqueline Hoare

**Affiliations:** 1 Department of Psychiatry and Mental Health, Faculty of Health Sciences, University of Cape Town, Cape Town, South Africa

**Keywords:** loneliness, burnout, doctors, mental health, health care

## Abstract

**Background:**

Loneliness is a subjective experience of emotional distress where individuals feel a lack of meaningful relational connections, and burnout results from chronic workplace stress. Doctors may be at increased risk, but there is a lack of data on the experience of loneliness and burnout among doctors in South Africa.

**Aim:**

This study aimed to assess the prevalence of loneliness, socio-demographic factors associated with loneliness and the association of loneliness with burnout in doctors working in a public academic hospital.

**Setting:**

Groote Schuur Hospital, Cape Town, South Africa.

**Methods:**

A cross-sectional quantitative survey study was conducted with doctors of all grades. Validated scales for assessing loneliness (UCLA Loneliness Scale) and burnout (Maslach Burnout Inventory) were administered via the survey.

**Results:**

Of 188 doctors, 71% reported moderate to high loneliness. A statistically significant correlation was found between UCLA loneliness and MBI scores. Unmarried doctors were lonelier, and those without children experienced higher burnout. Loneliness and burnout risk were associated with doctors who were older, more experienced and those working longer hours.

**Conclusion:**

There is a high prevalence of both loneliness and burnout in doctors working in a public academic hospital in Cape Town.

**Contribution:**

Interventions aimed at improving burnout in public academic hospital settings may benefit from strategies to enhance social connections and belonging in our institutions.

## Introduction

Loneliness has long been a theme in literature, but academic coverage of loneliness was sparse until recent years.^1^ More recently, alarms have been raised that loneliness may be a public health crisis.^1^ However, some critics argue that loneliness has not increased, but rather only academic focus on the topic has gained popularity.^1^ Loneliness can be described as a state of subjective emotional distress wherein individuals lack meaningful relationships regardless of their social network.^2^ Burnout, on the other hand, is a syndrome resulting from chronic workplace stress, and it is characterised by emotional exhaustion, cynicism related to work and reduced professional efficacy. International research in the general population has reported on the concerning impact of loneliness on physical and mental health outcomes.^2,3,4,5^ Previous meta-analytic reviews on the general population have highlighted how loneliness is linked to elevated mortality rates, with its effect comparable to established risk factors such as obesity, physical inactivity and smoking.^2,6^ Studies have also demonstrated associations between loneliness and the development of coronary heart disease, stroke, increases in systolic blood pressure and chronic pain.^6,7,8^ Individuals experiencing loneliness face heightened risks of anxiety, depression, dementia and suicide.^2,7,8,9^

According to World Health Organization, 30% of the total number South African doctors are responsible for caring for 85% of the population in the public sector, while the remainder of doctors work in the private health care system.^10^ In a 2018 survey of 1400 practicing United States (US) physicians, 25% of physicians reported feeling lonely and isolated, despite being in the constant company of others.^2^ There are limited data on the prevalence of loneliness and burnout in doctors working in South Africa despite challenges in the public sector such as resource constraints and understaffing.^3,11,12^ These constraints often lead to detrimental effects on the well-being of medical professionals.^3,4,5^

Loneliness may be particularly concerning in healthcare, where there are intense pressures to perform, and vulnerability is frequently discouraged.^2^ Despite their constant interactions with patients, doctors are susceptible to feelings of loneliness.^13,14^ Numerous factors, including workplace conditions, cultural norms and individual characteristics, contribute to loneliness within healthcare settings.^2,13^ Competitiveness and ambition within the field may hinder the ability to forge meaningful connections with colleagues and create a collaborative culture.^7^ Furthermore, the culture of self-sacrifice prevalent in medicine discourages self-care, leading to physical and emotional exhaustion.^13^ Doctors often hesitate to talk about their fears, the daily challenges they confront and the mistakes they make. They are often confronted by feelings of shame, guilt and self-stigmatisation.^7,13^ This can erode their ability to develop and sustain healthy professional relationships that further isolates them and exacerbates feelings of loneliness.^7,13^ The rigorous demands of clinical training consume a significant amount of time, separating doctors from social activities and life experiences shared, thereby exacerbating feelings of loneliness and risk of burnout.^13^ Loneliness can increase the risk of lower work quality as well absenteeism, impacting patient outcomes negatively.^2^ Loneliness and burnout together have been recognised to be associated with increased medical errors, covering up errors and lower patient satisfaction.^15^

The aim of this research study was to explore loneliness and burnout among doctors in a public academic hospital in South Africa and the potential associations of loneliness with burnout risk. The research objectives were: (1) to assess the prevalence of loneliness and burnout risk among doctors of all grades in a public teaching hospital setting; (2) to explore the association between socio-demographic factors and loneliness as well as burnout scores and (3) to identify any association between loneliness and burnout risk.

We hypothesised that:

- There would be a high prevalence of loneliness and burnout risk among doctors in a public academic hospital.
- There would be a significant association between loneliness and burnout risk.

## Research methods and design

The study was a cross-sectional survey study conducted at Groote Schuur Hospital (GSH). Groote Schuur Hospital is one of Cape Town’s tertiary public academic hospitals and serves as the primary academic hospital for the University of Cape Town’s medical school and employs 447 doctors. Groote Schuur Hospital delivers tertiary care across all major medical disciplines. The study participants included medical doctors of all grades who are currently employed at GSH, including interns, community service doctors, medical officers, registrars, consultants, as well as those involved in research projects conducted at GSH. The sample size was calculated using the Cochran formula with a confidence interval of 95%, and a margin of error of 10% was assumed. From the Cochran formula, out of 447 doctors at GSH, an acceptable response rate of 80 doctors was estimated.

### Measures

The socio-demographic factors collected in the survey were age, gender, self-identified group, marital status, number of children, country of origin, current department, grade of doctor, number of years in service and average weekly working hours. The survey also included whether the participants felt supported by their respective departments. In addition, the survey included the University of California Los Angeles (UCLA) Loneliness Scale-Version 3 used to assess loneliness.^16,17,18^ The UCLA loneliness score ranges from 20 to 80 with scores of 20–34 suggestive of a low degree of loneliness, 35–49 a moderate degree of loneliness, 50–64 a moderately high degree of loneliness and 65–80 a high degree of loneliness. Studies have confirmed the internal consistency, test-retest reliability and validity of the UCLA Loneliness Scale version 3.^16,17,18^ Research conducted in South Africa offers additional evidence of the scale’s applicability in local settings.^19^

Burnout risk was measured using the Maslach Burnout Inventory-Human Services Survey (MBI-HSS), which consists of 22 items and has a firmly established clinical validity.^20,21,22^ The MBI-HSS scales are emotional exhaustion, depersonalisation and personal accomplishment. Emotional exhaustion (EE) measures feelings of emotional and physical fatigue because of work-related stress; depersonalisation (DP) measures loss of empathy and emotional detachment in professional interactions and personal accomplishment (PA) assessment measures reduced job satisfaction and feelings of inefficacy. Scores of over 30 for emotional exhaustion and over 12 for depersonalisation, as well as scores of less than 33 for personal accomplishment, indicate higher risk of burnout (Online Appendix 1, Online Appendix 2).^22^

### Data collection

The anonymised survey was created using SurveyMonkey. Emails containing the link to the survey were initially sent to doctors via their Head of Departments with an explanation of the study and the institutional approval letters. Additionally, QR codes to the survey link were printed and distributed across departments at GSH. The initial form to complete in the survey was the consent form, which explained the nature and purpose of the study, as well as the confidentiality of their responses. The completed questionnaires were automatically directed to the researcher’s secure e-mail address; the completed emailed questionnaires did not include any names or e-mail addresses. The collected data were stored in a password-controlled file to further protect participant identity.

### Data analysis

Data collected from the questionnaires were analysed using the SPSS software, version 30.0. Descriptive statistics were used to describe the profile of the participants. Pearson’s correlation analysis was employed to study the association between loneliness and burnout. For significance, a *p*-value of less than 0.05 indicated statistical significance. Regression analysis was used to determine the relationship between socio-demographic variables and loneliness as well as burnout. Simple linear regression was employed to compare the dependent and independent variables.

### Ethical considerations

Ethics approval for this study was obtained from the Human Research Ethics Committee (HREC) UCT Faculty of Health Sciences on 15th October 2024 (HREC: 575/2024) (Online Appendix 3, Online Appendix 4, Online Appendix 5). The research conformed to the recommendations of the Declaration of Helsinki. Institutional permission to conduct this study was obtained from GSH on 8th November 2024. Respondents were informed that they have the right to decline participation or to withdraw from the study at any time they wish. Informed consent was completed at the beginning of the survey. All data were kept secure and made available only to members of the research team.

## Results

Two hundred and fifteen doctors participated in the survey between 13 November 2024 and 26 January 2025, of whom 58.1% identified as female, 41.4% male and 0.5% identifying as other. One hundred eighty-eight participants completed both the loneliness and MBI scales. The respondents’ ages ranged from 24 years to 76 years, with a mean of 35.78 years and the majority between 31 years and 40 years (50.2%). Fifty-two percent (52.6%) of respondents were married, and 63.8% did not have children (Table 1). The largest proportion of respondents had 5–10 years of experience (32.6%), followed by 11–20 years (29.3%). In terms of self-identified population groups, white/Caucasian people (49.8%) was the largest group, followed by black African people (18.1%), Indian people (15.3%) and Coloured people (13.5%). The majority (80.5%) were born in South Africa.

**TABLE 1:** Demographic characteristics of respondents (*N* = 215).

Variable	*n*	%
**Gender**		
Female	125	58.1
Male	89	41.4
Other (specify)	1	.5
**Age group (years)**		
Up to 30	63	29.3
31–40	108	50.2
41–50	22	10.2
51–60	15	7.0
> 60	7	3.3
**Marital status**		
Yes	113	52.6
No	102	47.4
**Parenthood**		
Yes	78	36.3
No	137	63.8
**Years of experience**		
< 5	47	21.9
5–10	70	32.6
11–20	63	29.3
> 20	35	16.3
**Self-identified group**		
Black people	39	18.1
Coloured people	29	13.5
Indian people	33	15.3
**Multiple ethnicity/Other**	7	3.3
White/Caucasian people	107	49.8
**Country of Birth**		
South Africa	173	80.5
Outside South Africa	42	19.5

Note: Registrars represented the largest group of respondents (46.05%), followed by consultants (20.47%), interns (17.21%) and medical officers (12.09%).

The departments with the highest number of respondents were Psychiatry (22.8%), Medicine (19.7%), Surgery (8.8%) and Anaesthesia (8.8%). Most respondents worked 50–60 h per week (36.3%), while 26.5% worked 60–70 h, and 9.3% worked over 80 h weekly. While 58.6% of respondents felt supported by their department, 41.4% did not feel supported. Data from Figure 1 show that moderate loneliness (38.3%) was the most common experience among respondents. High loneliness (32.7%) also affected a significant proportion, and very high loneliness was reported by 8.7% of participants and low loneliness by 20.4% of respondents.

**FIGURE 1:**
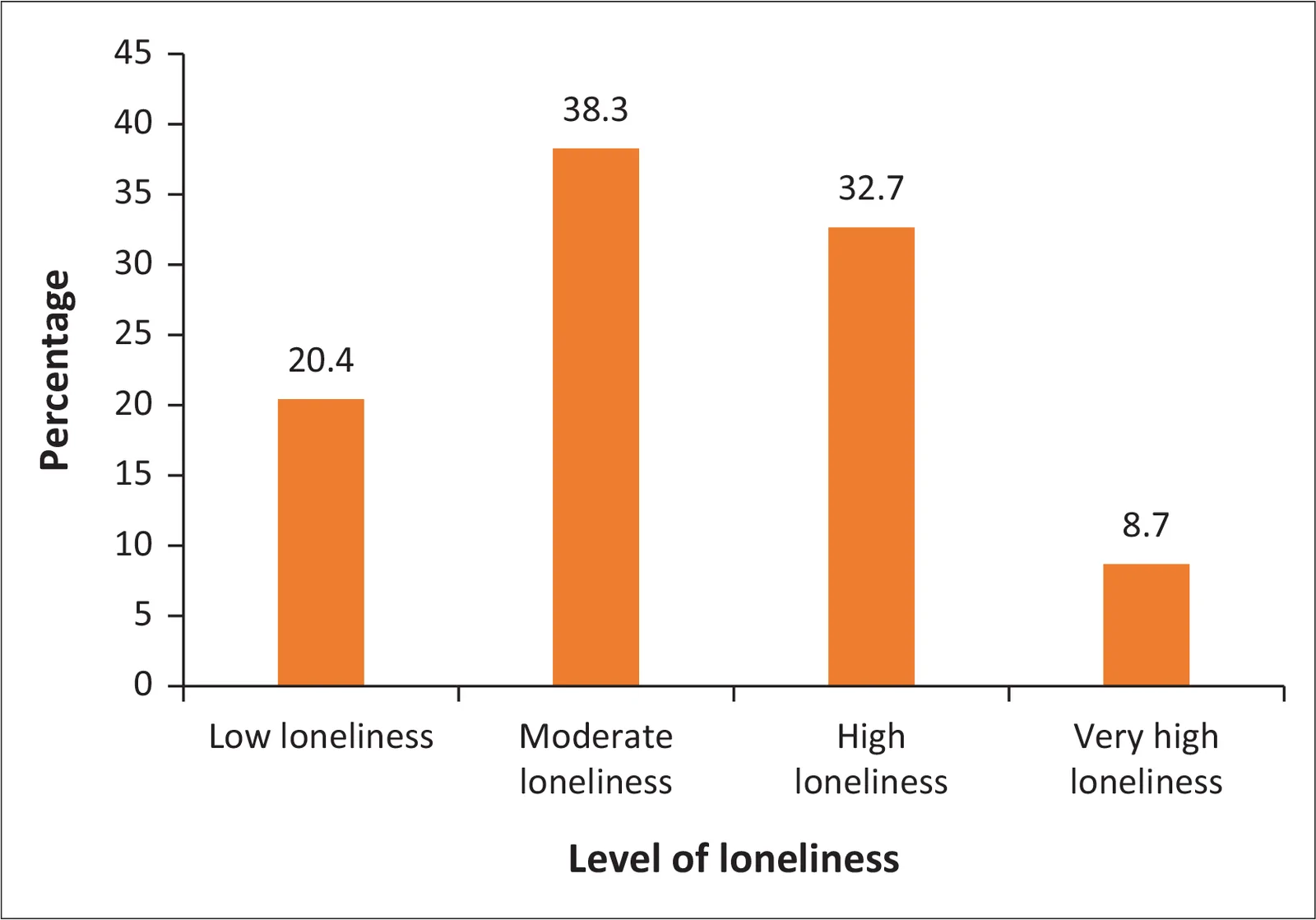
Loneliness score and percentage of respondents in each category.

50.8% of respondents (*n* = 97) reported a high degree of exhaustion, 26.7% of respondents (*n* = 51) experienced moderate exhaustion and 22.5% of respondents (*n* = 43) reported low exhaustion.

48.4% (*n* = 93) reported a high degree of DP, 29.2% of respondents (*n* = 56) had moderate DP and 22.4% of respondents (*n* = 43) reported low DP.

69.1% of respondents (*n* = 132) reported a low sense of PA, 18.8% of respondents (*n* = 36) reported a moderate sense of accomplishment and 12.0% of respondents (*n* = 23) reported a high sense of PA. A statistically significant correlation (0.309, *p* < 0.01) was found between loneliness and burnout scores.

No statistically significant difference was noted in loneliness (*p* = 0.538) and burnout scores (*p* = 0.791) between males and females. Unmarried doctors reported higher loneliness (*p* = 0.014) compared to married doctors (Table 2).

**TABLE 2:** Gender, marital status and parenthood statistics.

Characteristic	Category	*n*	Loneliness Mean ± s.d.	*p*-value	Burnout Mean ± s.d.	*n*	*p*-value
Gender	Male	83	2.28 ± 0.59	0.538	3.17 ± 0.82	82	0.791
	Female	112	2.34 ± 0.63	-	3.20 ± 0.72	107	-
Marital status	Married	100	2.22 ± 0.66	0.014	3.10 ± 0.82	98	0.078
	Unmarried	96	2.43 ± 0.54	-	3.29 ± 0.69	92	-
Parenthood status	Has children	73	2.24 ± 0.62	0.190	2.96 ± 0.77	122	0.001
	No children	122	2.36 ± 0.60	-	3.32 ± 0.73	119	-

s.d., standard deviation.

There was no statistically significant difference in loneliness scores between those who had/did not have children (*p* = 0.190). Those without children reported significantly higher burnout scores, however (*p* = 0.001).

Significant differences were found between those working 60–70 h, who reported higher loneliness (*p* = 0.013) and burnout scores (*p* = 0.023) than those working 40–50 h. Even those working over 80 h reported significantly higher loneliness compared to those working 40–50 h or 50–60 h (*p* = 0.003).

Older individuals (> 60 years) reported higher loneliness than those under 30 years (*p* = 0.027), those aged 31–40 years (*p* = 0.008) and those in the 41–50 years category (*p* = 0.031). They also had higher burnout scores than those under 30 (*p* = 0.020). In terms of years of experience, no significant differences were found between any experience groups (*p* > 0.05) for loneliness. Individuals with more than 20 years of experience reported significantly higher burnout (*p* = 0.008) than those with less than 5 years of experience.

No statistically significant difference in loneliness (*p* = 0.604) or burnout scores (*p* = 0.455) was noted across departments (Online Appendix 6).

## Discussion

The current study is the first to report on loneliness and its correlation with burnout risk in a public academic hospital in South Africa. Firstly, we found a statistically significant correlation between loneliness and burnout scores (*p* < 0.01). Secondly, we found that a higher than expected (48%) percentage of doctors participated in the survey, and 42% completed both scales. This is suggestive of an awareness and interest in highlighting loneliness and burnout as issues of concern. Thirdly, we found that 38.3% of respondents were experiencing moderate and 32.7% high loneliness. Fourthly, we found that doctors reported a high degree of exhaustion (50.8%), DP (48.4%) and low PA (69.1%), suggestive of risk of burnout. Fifthly, we found that unmarried doctors were lonelier (*p* = 0.014) and those without any children experienced higher burnout risk (*p* = 0.001). Doctors who were older and worked longer hours reported being lonelier and were more likely to be burnt out than others. Those who were more experienced reported more burnout scores than their peers (*p* = 0.008).

The current study confirms our hypotheses of a high prevalence of loneliness and burnout risk among doctors working in a public academic hospital in South Africa, as well as the significant relationship between loneliness and burnout risk. While the data reported on loneliness and burnout is alarming, it does correspond to previous research findings in international studies. A US study carried out during the coronavirus disease (COVID) pandemic had 48% of the respondents reporting feeling lonely.^2^ In another US study on loneliness in physicians in pre-COVID times, 18.0% met the criteria for intense loneliness.^23^ Research on family medicine physicians in US also showed a loneliness and burnout prevalence of 44.9% and 45.1%, respectively.^15^ The relationship between loneliness and burnout has rarely been studied, but the bidirectional relationship between the two has been described in international studies.^2^ Previous research has also reported that doctors with burnout were more likely to report feeling lonely than those without significant burnout.^15,23,24,25,26^ Emotional exhaustion is characterised by depletion of energy because of being overwhelmed by work as well as a lack of motivation to engage with people and the tasks of everyday life.^22^ Connecting meaningfully with others and belonging in an institution takes a particular level of effort and energy that is likely to be lacking when doctors are emotionally exhausted.^2,22^

While the current study found no significant gender differences in loneliness, higher prevalences of loneliness has been noted previously among female health care workers.^15,23^ Studies in US physicians and residents had attributed the higher prevalence in females to challenges such as balancing a demanding career and family time.^15,23^ Another contrasting finding in the current study was how South African and international doctors working in South Africa appeared equally lonely and more likely to be burnt out. In previous studies among US doctors, positive correlations between burnout and social loneliness were found in those living away from their families and friends.^25,27^ Increased support from family and friends has been found to reduce loneliness and burnout among doctors.^25^

Older and more experienced doctors reported more loneliness and burnout than younger colleagues. In contrast to our findings, prior studies have found doctors with more years of practice to have lower levels of loneliness.^15,23^ Those findings have been attributed to better acclimatisation and an efficient work–life balance, and older doctors were also more likely to be married and have children, which was considered protective.^15,23^

In the South African context, many of the experienced doctors in public care would have faced the worst of the human immunodeficiency virus/acquired immunodeficiency syndrome (HIV/AIDS) epidemic prior to the availability of antiretrovirals in the 1990s and 2000s. Younger doctors are faced with numerous challenges but have to face less resource constraints than their older peers. The COVID-19 pandemic also exacerbated the strain on South Africa’s already overwhelmed healthcare system, increasing the physical and mental health risks faced by medical practitioners.^3,28^ More recently, the 2024/2025 National Health budget cuts have resulted in an additional impact on the mental health of the senior doctors who have to make morally distressing decisions on service delivery. These might explain why senior doctors are more exposed to loneliness and risk of burnout.

### Limitations

The study was conducted in one academic hospital affiliated to the University of Cape Town (UCT) despite the initial proposal of including four different hospitals; it was the result of administrative challenges and time constraints. It should be noted that GSH is a tertiary centre and the results could differ from other hospitals; the urban location might also affect generalisability of results to the rest of the province or the country.

Participation from the different departments was varied, which detracts from an accurate comparison across units at GSH. It should also be noted that most participants were under 40 years of age and had less than 20 years of experience. Despite measures in place to minimise bias, self-selection bias might have occurred as participants can voluntarily decide about their response to the research.

Another limitation is the lack of South African literature on loneliness among doctors. Most of the research mentioned was conducted in the US, where health system resources and burden of disease are different.

## Conclusion

This study highlights a concerning level of loneliness in doctors working at a public academic hospital in Cape Town as well as the relationship between loneliness and burnout risk. Workplace challenges appear to contribute more towards loneliness than other factors like gender. While a career in medicine can be deeply fulfilling, it also presents considerable challenges, and medical professionals are disproportionately susceptible to mental health issues compared to the general population.^14,29^ Recent budget cuts and understaffing might have exacerbated the situation. Other explanations might include the burden of disease in South Africa and the ongoing exodus of medical personnel. Groote Schuur Hospital launched a staff wellness centre in 2022, which is dedicated towards providing support to its staff. Nearly 60% of participants felt supported by their departments, but they reported high degrees of loneliness. The findings from this study highlight the need for further programmes or restructured work environments to reduce loneliness and enhance the well-being of doctors. Initiatives to prevent and reduce loneliness and burnout among doctors should focus on increasing opportunities for meaningful social interactions at work. Weekly peer support sessions would enable doctors to open up and improve their well-being.^7^ It is possible to improve the experience of our healthcare workforce through organisational and personal solutions.^2^ It is also hoped that occupational health and wellness will be at the heart of open debates and discussions.

Further research is warranted in other hospitals to compare the prevalence of loneliness; it would also be recommended that similar studies be carried out with allied health professionals, such as psychologists, physiotherapists, speech therapists, occupational therapists, etc. Other healthcare staff at GSH expressed significant interest in the loneliness research during data collection. As GSH is a tertiary centre, it is encouraged that research be conducted in smaller settings, rural areas and community clinics as well.
